# Case of a Man, Whose Body Was Covered with Encysted Tumours

**Published:** 1785

**Authors:** 


III. *Case of a man, whose body was covered with encysted tumours. By Mr.* John O Donnel, *Surgeon at Chelmsford, in Essex.*

THOMAS Barker, of Clavering, in Essex, aged thirty five years, about eight years ago perceived a small tumor of the wen kind under his chin, to which he paid no regard. Shortly after, another of the same kind appeared at the back of his neck; and in a very little time more, both his arms, from the shoulders to the wrists, were affected in like manner. Gradually the trunk and lower extremities became affected in the same way, and much distorted.

At present the wen at the chin is about the size of a child's head of eight years old, and of a conical figure, with its basis upwards. His arms are prodigiously enlarged. The integuments of the thorax are in a half inflated state, but divided at the sternum; so that they appear like two distinct bags, or bladders, half filled with a thin fluid. The abdomen from the navel downwards is one continued tumour, so flaccid and distended as to hang half way down the thighs.—This he complains of as a great great inconvenience, for if, in the night time, he has occafion to make water, he is obliged to hold it up with his hand.—In the day time it is fuftained by his breeches.—His back is in a fimilar ftate with the abdomen ; each fide of it, from the neck downwards, being one tumor ; fo that when he lies on one fide, the tumor on the other laps over the fpine which divides them. The fcrotum, at its moft dependent part, and the perinæum are fo tumefied as to have the appearance, when his body is erect, of a fecond fcrotum. In fhort, all parts of his body (his hands, feet, penis, and the integuments of the cranium excepted), are in fo uncommon a degree diftorted, that, when naked, he fcarcely appears human.

He was naturally a thin fpare man (never in his life weighing above nine ftone), with a good ftate of health, of a lively temper, and not ufed to hard labour.

At the firft appearance of thefe wens, and for fome years before, he drank very freely, particularly of fpirituous liquors.—His breakfaft generally confifted of beef fteaks and ftrong beer, and his fuppers, alfo, were of animal food, and always plentifully diluted with ftrong beer, or gin and water, but more frequently quently with the latter. He was of a very lax habit of body, having, from his childhood, generally had three or four ftools a day, and frequently many more. Ever fince the firft appearance of thefe excrefcences he has found his breathing affected when confined to his room for any length of time, but on removing to the open air he breathes freely again.

For nine months paft he has been confined as a debtor in Chelmsford gaol, and during great part of this time has been troubled with phthifis pulmonalis, and is gradually growing worfe.—At prefent he is in the laft ftage of this fatal difeafe.

His wife has five children by him, all healthy and ftrong; of thefe, four were born before the wens appeared, and one after. The firft four were born within the term of five years, and the laft feven years after; which was fix years after the wens appeared.

P. S. I took the above account from himfelf and his wife on the 16th of laft month, fince which time he died;—but I could not prevail on his wife to permit me to open any of the tumors. They were all of that kind called meliceris, though many of them felt as if they contained a fluid not thicker than ferum.

*Chelmsford,*
*Feb. 11, 1785.*

